# Environmental Influences on Children's Physical Activity: Quantitative Estimates Using a Twin Design

**DOI:** 10.1371/journal.pone.0010110

**Published:** 2010-04-21

**Authors:** Abigail Fisher, Cornelia H. M. van Jaarsveld, Clare H. Llewellyn, Jane Wardle

**Affiliations:** Department of Epidemiology and Public Health, Cancer Research UK Health Behaviour Research Centre, University College London, London, United Kingdom; Karolinska Institutet, Sweden

## Abstract

**Background:**

Twin studies offer a ‘natural experiment’ that can estimate the magnitude of environmental and genetic effects on a target phenotype. We hypothesised that fidgetiness and enjoyment of activity would be heritable but that objectively-measured daily activity would show a strong shared environmental effect.

**Methodology/Principal Findings:**

In a sample of 9–12 year-old same-sex twin pairs (234 individuals; 57 MZ, 60 DZ pairs) we assessed three dimensions of physical activity: i) objectively-measured physical activity using accelerometry, ii) ‘fidgetiness’ using a standard psychometric scale, and iii) enjoyment of physical activity from both parent ratings and children's self-reports. Shared environment effects explained the majority (73%) of the variance in objectively-measured total physical activity (95% confidence intervals (CI): 0.63–0.81) with a smaller unshared environmental effect (27%; CI: 0.19–0.37) and no significant genetic effect. In contrast, fidgetiness was primarily under genetic control, with additive genetic effects explaining 75% (CI: 62–84%) of the variance, as was parent's report of children's enjoyment of low 74% (CI: 61–82%), medium 80% (CI: 71–86%), and high impact activity (85%; CI: 78–90%), and children's expressed activity preferences (60%, CI: 42–72%).

**Conclusions:**

Consistent with our hypothesis, the shared environment was the dominant influence on children's day-to-day activity levels. This finding gives a strong impetus to research into the specific environmental characteristics influencing children's activity, and supports the value of interventions focused on home or school environments.

## Introduction

Contemporary models identify biological (genetic) factors [1], deliberate health-related choices (behaviour) [2], and features of the home and local environment [3] as important determinants of physical activity. Environmental factors attract particular attention because they can be targeted by policy or planning [4]. However, it has proved difficult to quantify the environmental contribution from ecological studies because the circumstances in which people live or work are partly endogenously determined. The gold standard design would modify the environment and examine effects on activity, but existing environmental intervention studies in children, most of which focus on the school context, have produced mixed results [5]–[8]; in part because of the difficulty of targeting the key environmental drivers. This has led to calls for novel research designs that can quantify contribution of environmental influences to physical activity [4].

Twin studies offer a type of ‘natural experiment’ that can estimate the contribution of environmental factors to variation in the target phenotype while controlling for genetic differences between individuals. Insofar as monozygotic (MZ) twin pairs are more similar in activity levels than dizygotic (DZ) twins, this implicates genetic influence, while the remainder of the variability can be attributed to environmental influence [9]; [10]. The total environmental effect includes shared and unique components. The shared environment effect (i.e. environmental influences that make family members more alike) is estimated from the extent to which twins are more similar than would be predicted from the genetic contribution to activity levels. The remaining variance is attributed to unique environmental factors that make family members different from one another plus error of measurement [9]; [10]. In the paediatric context, evidence for a strong shared environment effect would support the value of home and school-based interventions.

There have been several twin studies of self-reported leisure time activity in adults, of which the largest used data from seven European twin registries (GenomEUtwin) with a total of over 37,000 twin pairs [11]. Using a cut-point of 60 minutes of activity at ≥4 METS a week to identify exercisers, heritability estimates ranged from 48% to 71%, with little or no evidence of any shared environment effect [11]. However, a recent study from a smaller North American twin registry hypothesised that a more stringent definition of being an exerciser (based on the CDC recommendation of 150 minutes a week for health benefits) would show a stronger environmental effect, because activity at that level is likely to be due to deliberate health-related decisions, which would in turn be influenced by environmental opportunities and barriers. They replicated GenomEUtwin's heritability estimate using the 60 minute cut-off, but as predicted, found no significant heritability for the 150 minute indicator; the best-fitting model included only shared (28%) and unique (72%) environmental influences [12].

Estimates of genetic and environmental effects on physical activity generated from adult studies cannot be assumed to generalise to children because the genetic contribution may show increasing expression with age [13]. Effects of the early shared environment might also be diluted with the passage of time. Adult twin studies are therefore likely to underestimate the shared environment effect on children's activity levels.

Studies of generalised motor activity in infancy [14] and early childhood [15] typically find evidence for significant heritability whether using parental reports [16] or objective measures [14]; [15], but it is uncertain whether infant motor activity is analogous to physical activity later in childhood; it may be more like fidgeting [17]. A small number of twin studies have used objective measures to examine heritability of physical activity level in children, but results have been equivocal. Plomin and Foch (1980) found extremely high intraclass correlations for pedometer counts in 30 MZ and 18 DZ twin pairs (r = .99, r = 0.94) aged 5–12 years, indicating no heritable influence on one week step-count [18]. Another study (38 DZ pairs, 62 MZ pairs, aged 4–10 years) measured total energy expenditure with doubly-labelled water (DLW) to calculate habitual physical activity energy expenditure (PAEE) [19]. Twin analyses showed no evidence for a heritable component, and the majority of the variation (69%) in PAEE was attributable to the shared environment. In contrast, a study of activity levels measured with accelerometery during an individual 2.5-hour testing session in a psychological laboratory found moderate heritability (around 36%) and a mixture of shared and non-shared environmental effects [20]. One explanation for the different results could be that in the laboratory testing session children were given the opportunity for free-play, and therefore subtle genetic differences in activity choices could be expressed, whereas in the normal life context assessed in the PAEE and pedometer studies, the constraints and demands of family and school life (the shared environment) have an overriding influence.

The purpose of the present study was to compare environmental and genetic estimates for three indicators of physical activity: i) objectively measured habitual physical activity (using accelerometry) from which we generated measures of total activity, moderate and vigorous activity (MVPA) and sedentary time, ii) fidgetiness (using a standard psychometric scale completed by parents), and iii) enjoyment of activity based on both parent ratings and children's self-reported activity preferences, in a sample of 9-12 year-old twin pairs (n = 234 children). We predicted that fidgetiness and enjoyment of activity would be heritable, but that objectively-measured daily activity would show a strong shared environment effect.

## Methods

### Participants

Participants were drawn from the Twins Early Development Study (TEDS), a twin birth cohort that originally included 16,000 families with twins born in England in 1994-96; representing more than half of all twins born during that period [21]. A subsample of 214 families with same-sex twin pairs, half with obese and half with lean parents, were selected for an intensive study of appetite and growth when the children were aged 4–5 years [22]. Three quarters of these families (n = 161) were followed up when the children were aged 9–12 years and measures of fidgetiness, activity preferences and objectively measured activity were taken at this time. Ethical approval was granted by the University College London Committee for the Ethics of non-NHS Human Research. Verbal consent was obtained from parents on the telephone before the home visit, and consent forms were completed by the parents on the day of the visit.

### Objectively measured activity

Activity was measured using the Actigraph model 7164 accelerometer (formerly the CSA/MTI) which is the most commonly used device in paediatric physical activity research, and has the most evidence of validity in children [23]. Although validation data were not collected in this sample, the Actigraph correlates well with PAEE by DLW in children of a similar age to the ones studied here [24]. Children were asked to wear the monitor on the right hip for seven consecutive days, removing it only for swimming, bathing and sleeping. Monitors were set to record data in one-minute epochs. Actigraph data files were processed using the MAHUffe programme (www.mrc-epid.cam.ac.uk/Research/PA/Downloads.html). Outcomes examined in the current study were total physical activity (total PA; mean accelerometer counts per minute), time spent in MVPA (>2000 counts per minute) [25], and time spent sedentary (<100 counts per minute). To distinguish between sedentary behaviour and time where the monitor was not worn, periods of data with more than 10 consecutive zeros were excluded [26]. It appeared that some children had worn the monitor throughout the night, so only valid data between the hours of 7am and 10pm were included in the analyses for all children. Data were collected from both twins in the pair at the same time. Timing of activity measurements for MZ (June 2005–June 2006) and DZ twins (March 2005–June 2006) were similar.

Physical activity data were obtained successfully from 234 children (57 MZ and 60 DZ same-sex twin pairs). Missing data were as follows: 38 children provided fewer than the three days with at least 600 minutes per day of data required to accurately represent children's usual activity [26]; [27], 32 accelerometers had technical errors and generated an invalid data download, and two accelerometers were lost. Data from an additional 14 children were excluded because data were missing for their co-twin. There were no significant differences in age, gender, body mass index standard deviation score (BMI SDS), socioeconomic status (SES) or ethnicity between those who provided valid activity data and those who did not (p values all >0.05).

### Fidgetiness

Fidgetiness was assessed using questions from a validated psychometric scale, the Conner's Rating Scale, that has been standardised in UK school-aged children [28]. Parents rated their children on four items (e.g. ‘my child has difficulty staying seated’) using a five-point likert scales (never; rarely; some of the time; most of the time; always). Scores used in these analyses were the mean of these four items, with higher scores indicating greater fidgetiness.

### Enjoyment of activity

Mothers provided ratings of their children's enjoyment of a selection of pastimes (my child enjoys … bike riding, walking, paddling, playing ball, etc.) on four-point likert scales from ‘not at all’ to ‘loves it’ (adapted from [29]). Activities were grouped into ‘low impact’ (e.g. board games), ‘medium impact’ (e.g. walking) and ‘high impact’ (e.g. running), and a mean score taken for each level. Higher scores indicate greater enjoyment. These scales have shown moderate tracking from 4 to 11 years, and have been shown to be associated with objectively measured activity in this sample [30]. Children's own stated activity preferences were assessed using a series of discrete choices from 48 pairs of more *vs* less active pastimes, with higher scores representing preferences for more active pastimes.

### Anthropometric and demographic data

At the home visit, trained researchers measured children's weights to the nearest 0.1 kg using Tanita scales and heights using a portable stadiometer. Body mass index (BMI) was calculated and converted to age-and-gender appropriate standard deviation scores (BMI SDS) relative to 1990 reference data [31]. Parents reported the twins' ethnic origin from one of four options: asian, black, mixed race, white. Family socioeconomic background (SES) was indexed by the mother's educational level (range 1-8, higher scores indicate higher SES). Zygosity information was obtained from a parent-reported questionnaire which is 95% reliable when compared to zygosity assigned by DNA in the TEDS sample [32]; pairs where zygosity was uncertain based on questionnaire results (about 5%) were diagnosed using DNA.

### Statistical analysis

Twin correlations can be inflated due to gender and age matches, so the data were regressed on these variables and residualized scores used in all analyses. Within-pair intraclass correlations were calculated for all physical activity variables. ACE model-fitting analyses were carried out using standard structural equation modelling in MX software (version 32; Virginia Commonwealth University, Richmond, VA). The ACE model fits the variance into additive genetic variance (a^2^), common environment (c^2^) and unique environment and measurement error (e^2^). MX software fits the full ACE model and sub-models to the data. Two fit indexes are recorded: chi-square and the Akiake Information Criterion (AIC). Sub-models were tested that were limited to CE, AE and E. The most parsimonious model was chosen as the model containing fewest parameters but with no significant worsening of fit compared with the full ACE model.

## Results

Participant characteristics stratified by zygosity are shown in **Table 1** **.** DZ twins were slightly older than MZ twins (p = 0.02) but there were no other significant differences in socio-demographic or physical activity variables (p values all >0.05).

**Table 1:** Baseline characteristics of twins stratified by zygosity.

	Monozygotic (n = 57 pairs)	Dizygotic (n = 60 pairs)
Age in years	11.06 (0.59)	11.27 (0.48)*
BMI	18.81 (3.27)	18.90 (3.53)
BMI SDS	0.43 (1.18)	0.40 (1.13)
Mother's education level (SES)	3.77 (1.95)	3.63 (1.90)
Ethnicity coded as ‘white’ (%)	93	95
Sex (% male)	46	46
Total physical activity	696.12 (216.30)	657.69 (228.17)
Time spent in MVPA in minutes	64.94 (31.92)	57.09 (31.32)
Number of valid† days accelerometer worn	6.0 (1.4)	6.0 (1.5)
Time spent sedentary in minutes	284.97 (73.07)	287.55 (68.56)
Fidgetiness score	2.15 (0.77)	2.48 (0.88)
Enjoyment of low impact activity	2.98 (0.48)	2.86 (0.53)
Enjoyment of medium impact activity	2.95 (0.59)	2.83 (0.59)
Enjoyment of high impact activity	3.01 (0.54)	2.76 (0.67)
Activity preference	0.26 (0.42)	0.27 (0.42)

Values are means and (SD) unless stated otherwise;

*significantly higher than MZ pairs (p<0.05); MVPA moderate and vigorous physical activity mean accelerometer >2000 counts per minute, sedentary time <100 counts per minute; Fidgetiness score, parent rated based on the scale by Taylor et al. (1984); Child's enjoyment of low, medium and high impact activity reported by parent; Activity preference calculated as mean of 48 activity choice (active vs sedentary activity) reported by child;

† ≥600 minutes of data.

Twin intraclass correlations for each physical activity measure are shown in **Table 2**. As predicted, MZ correlations were higher than DZ correlations for fidgetiness (0.71 vs 0.30), enjoyment of low, medium, and high activities (MZ: 0.70–0.84 vs DZ: 0.52–0.60) and activity preference score (0.59 vs 0.33), indicating genetic influence. In contrast, accelerometer counts for total PA and MVPA showed smaller MZ-DZ differences (total PA: 0.76 vs 0.71; MVPA: 0.69 vs 0.52), and sedentary time showed an intermediate effect (0.62 vs 0.48), suggesting environmental influence.

**Table 2:** Intraclass correlations for physical activity phenotypes.

Variable	Monozygotic (n = 57 pairs)	Dizygotic (n = 60 pairs)
Total physical activity	0.76 (0.62, 0.85)**	0.71 (0.56, 0.81)**
Time spent in MVPA in minutes	0.69 (0.53, 0.81)**	0.52 (0.31, 0.68)**
Time spent sedentary in minutes	0.62 (0.42, 0.75)**	0.48 (0.26, 0.65)**
Fidgetiness score	0.71 (0.59, 0.81)**	0.30 (0.10, 0.49)**
Enjoyment of low impact activity	0.70 (0.57, 0.79)**	0.52 (0.35, 0.65)**
Enjoyment of medium impact activity	0.80 (0.71, 0.87)**	0.60 (0.45, 0.72)**
Enjoyment of high impact activity	0.84 (0.77, 0.89)**	0.52 (0.35, 0.66)**
Activity preference	0.59 (0.39, 0.74)**	0.33 (0.10, 0.53)**

**p<0.001. Total physical activity (mean accelerometer counts per minute);

MVPA moderate and vigorous physical activity mean accelerometer >2000 counts per minute,

sedentary behaviour <100 counts per minute; Fidgetiness score, parent rated based on the scale by Taylor et al. (1984);

Child's enjoyment of low, medium and high impact activity reported by parent; Activity preference calculated as mean of 48 activity choice (active vs sedentary activity) reported by child…

Model-fitting analyses confirmed the impressions from the twin correlations. The best-fitting model for fidgetiness suggested high heritability (75%; 95% confidence interval (CI) 62–84%) with a contribution from the non-shared environment component of 25% (16–38%). Enjoyment of low (74%; CI 61–82%), medium (80%; CI 71–86%) and high (85%; CI 78–90%) impact activities and children's expressed activity preferences (60%; CI 42–72%) were also significantly heritable, with the non-shared environment explaining the remainder of the variance in each case (see **Table 3**). In contrast, the most parsimonious model for the objective measures of activity had no genetic contribution and strong shared environment effects, with the shared environment explaining 73% (63–81%) of the variance for total PA, 61% (48–71%) for MVPA and 55% (41–66%) for sedentary time. The non-shared environment explained 27% (19–37%) of the variance in total PA, 39% (30–52%) of MVPA, and 45% (34–59%) of the variance in sedentary behaviour (see **Table 4**).

**Table 3:** Genetic and environmental influences on fidgetiness, enjoyment of activity and child reported activity preferences in 9–12 year old twins.

Model	Estimates of variance components			Model fit				
	a^2^	c^2^	e^2^	-2LL	df	ΔAIC	Δ x^2^	p
Fidgetiness score								
ACE	0.75 (0.50, 0.84)	0.00 (0.00, 0.22)	0.25 (0.16, 0.40)	636.943	227	-	-	-
CE	-	0.47 (0.31, 0.60)	0.53 (0.40, 0.69)	653.971	228	15.028	17.028	<0.001
**AE** †	0.75 (0.62, 0.84)	-	0.25 (0.16, 0.38)	636.943	228	0	0	Incalculable
Enjoyment of low impact activity								
ACE	0.59 (0.19, 0.82)	0.15 (0.00, 0.48)	0.26 (0.18, 0.40)	614.546	227	-	-	-
CE	-	0.57 (0.44, 0.68)	0.43 (0.32, 0.56)	622.874	228	6.328	8.328	0.004
**AE** †	0.74 (0.61, 0.82)	-	0.26 (0.18, 0.39)	615.055	228	−1.491	0.509	0.476
Enjoyment of medium impact activity								
ACE	0.44 (0.14, 0.83)	0.35 (0.00, 0.62)	0.20 (0.14, 0.31)	600.975	227	-	-	-
CE	-	0.68 (0.57, 0.77)	0.32 (0.23, 0.43)	608.982	228	6.006	8.006	0.005
**AE** †	0.80 (0.71, 0.86)	-	0.20 (0.14, 0.29)	604.398	228	1.423	3.423	0.064
Enjoyment of high impact activity								
ACE	0.54 (0.27, 0.88)	0.32 (0.00, 0.57)	0.15 (0.10, 0.23)	567.275	227	-	-	-
CE	-	0.70 (0.60, 0.78)	0.30 (0.22, 0.40)	582.637	228	13.362	15.362	<0.001
**AE** †	0.85 (0.78, 0.90)	-	0.15 (0.10, 0.22)	570.503	228	1.229		0.072
Activity preference								
ACE	0.53 (0.01, 0.72)	0.06 (0.00, 0.47)	0.41 (0.28, 0.60)	628.833	227	-	-	-
CE	-	0.45 (0.30, 0.59)	0.55 (0.41, 0.70)	632.879	228	2.046	4.046	0.044
**AE** †	0.60 (0.42, 0.72)	-	0.40 (0.28, 0.58)	628.906	228	−1.927	0.073	0.786

Standard ACE model-fitting analyses were used to estimate additive genetic (a^2^), shared environment (c^2^), non-shared environment effects (e^2^).

† indicates the most parsimonious model; Fidgetiness score, parent rated based on the scale by Taylor et al. (1984); Child's enjoyment of low, medium and high impact activity reported by parent; Activity preference calculated as mean of 48 activity choice (active vs sedentary activity) reported by child MVPA moderate and vigorous physical activity mean accelerometer counts/min >2000; sedentary behaviour mean accelerometer counts/minute <100; LL log likelihood, AIC Akaike Information Criteria.

**Table 4:** Genetic and environmental influences on objectively measured habitual physical activity in 9-12 year old twins.

Model	Estimates of variance components			Model fit				
	a^2^	c^2^	e^2^	-2LL	df	ΔAIC	Δ x^2^	p
Total physical activity								
ACE	0.14 (0.00, 0.45)	0.63 (0.34, 0.80)	0.23 (0.15, 0.35)	497.837	227	-	-	-
**CE** †	-	0.73 (0.63, 0.81)	0.27 (0.19, 0.37)	498.901	228	−0.936	1.064	0.302
AE	0.78 (0.69, 0.85)	-	0.22 (0.15, 0.31)	510.988	228	11.151	13.151	<0.000
Time spent in MVPA in minutes								
ACE	0.28 (0.00, 0.73)	0.39 (0.00, 0.68)	0.33 (0.22, 0.48)	590.487	227	-	-	-
**CE** †	-	0.61 (0.48, 0.71)	0.39 (0.30, 0.52)	592.531	228	0.044	2.044	0.153
AE	0.69 (0.56, 0.79)	-	0.31 (0.21, 0.44)	593.991	228	1.504	3.504	0.061
Sedentary time in minutes								
ACE	0.24 (0.00, 0.69)	0.37 (0.00, 0.65)	0.39 (0.27, 0.57)	611.341	227	-	-	-
**CE** †	-	0.55 (0.41, 0.66)	0.45 (0.34, 0.59)	612.423	228	−0.918	1.082	0.298
AE	0.63 (0.48, 0.74)	-	0.37 (0.26, 0.52)	614.132	228	0.791	2.791	0.095

Standard ACE model-fitting analyses were used to estimate additive genetic (a^2^), shared environment (c^2^), non-shared environment effects (e^2^).

† indicates the most parsimonious model; Total physical activity (mean accelerometer counts per minute) MVPA moderate and vigorous physical activity mean accelerometer counts/min >2000; sedentary behaviour mean accelerometer counts/minute <100; LL log likelihood, AIC Akaike Information Criteria.

## Discussion

The results of this study indicate that children's fidgetiness and enjoyment of activity are under predominantly genetic control, whereas objectively-measured daily physical activity is influenced primarily by the shared environment.

Few studies have examined the heritability of fidgetiness in children using a twin design, although numerous animal studies have shown biological influence on spontaneous physical activity (see [33] for review). While it cannot be discounted that high heritability estimates are, to some extent, a product of parent-reporting bias, some studies support our findings. In the previously reported pedometer study, Plomin and Foch (1980) also reported intraclass correlations on directly-observed fidgeting that were indicative of genetic influence [18]. Wood et al. (2008) estimated high heritability (62%) on parent-reported symptoms of hyperactivity in 436 7–9 year old twins, with no shared environment effect [34]. Genetic influence has been supported in studies that show links between fidgetiness or generalised infant motor activity and endocrine function. In infants, the dopamine DRD4 receptor has been shown to be associated with activity temperament [35] and infants with the L-DRD4 receptor were found to be more active in a free play situation [35]. In adults, administration of a dopamine receptor antagonist reduced spontaneous motor activity level by 41% [36]. Non-exercise activity thermogenesis (NEAT), the energy expended during spontaneous motor activity [37], has already been implicated in weight gain in adults [38], and it would be useful to examine the relationship in children [39].

Children's expressed preferences and their parents' reports concerning enjoyment of activity also proved to be highly heritable in this study. Whether a child enjoys being active or not may be influenced by temperament or physical skills which themselves show genetic influences [16]; [40]. The dopamine system has been implicated in the liking and reinforcing value of physical activity [41], and genes relating to the dopamine system have been associated with physical activity levels in women [42]. Our findings on activity preferences may have a similar endocrinological origin. Liking for sedentary activities is higher in children at risk for overweight (with two overweight parents) [22], and there is evidence that overweight children find sedentary activities more reinforcing than active pastimes [43].

In contrast to the findings for fidgetiness or enjoyment of activity, we found that objectively-measured activity was entirely environmentally determined. This concurs with two previous studies examining heritability of daily activity in children [18]; [19]. The use of accelerometery in our study represents a significant advance in the paediatric literature examining genetic and environmental influences on activity, because it is a more direct measure of activity. The use of PAEE by DLW in twin studies, as by Franks et al. [19], can be problematic, because PAEE is highly correlated with body weight [44] making it difficult to untangle any genetic influence on activity from genetic influence on weight. However after PAEE was adjusted for weight in the study by Franks et al. the proportion of variance explained by shared environmental factors was remarkably similar to the variance observed in this study (69% *vs* 73%) [19]. These results are consistent with a recent systematic review which concluded that environmental interventions can increase children's physical activity, although there were few studies on younger children and some notable failures of apparently heroic interventions [5]. In further support of this Saudino & Zapfe (2008) reported no shared environmental influence on activity level in 2 year old twins in a laboratory, but a strong (56%) shared environment effect when the same twins were measured at home over 2 days [15]. It is possible that the longer the period of objective activity measurement, the more dominant the shared environmental effect becomes. In contrast to our study these authors did detect a genetic effect on activity level [15], however it is feasible that in younger children, as a larger proportion of their time is likely spent in ‘free play’ activities, subtle genetic effects can be more clearly detected. It is also possible that the environment restricts the natural activity patterns of children. This does not mean that genetics do not influence childhood physical activity in any circumstance, simply that the environment was the dominant influence on day-to-day activity in our study. There is some evidence that being in, for example, a school environment can substantially restrict activity levels in 8-13 year olds [45]. It is likely that children's behaviour is influenced strongly by parents and teachers, and where subtle genetic differences do exist, it is feasible that they are only expressed where the freedom to choose activities is available. Another potential explanation is that longer measurement periods reduce measurement error. Future research should aim to untangle these issues.

### Limitations

There were limitations in the current study. Firstly, inevitably, parental ratings introduce bias; for example where parents have a tendency to report MZ (identical) twins as more similar than DZ twins. In some previous twin studies where parent report has been used to assess infant motor activity level, reporting bias has been evident [46]; [47]. The differences in genetic estimates that we observed in this study, a focal point of our paper, could be influenced by this bias, which may overestimate the heritability of parent-reported outcomes. However in our study, twins also independently reported their own activity preferences, and significant heritability was still observed, providing some support for the parental data. Further, reporting bias in twin studies is usually evidenced by ‘too low’, or even negative, DZ correlations [47] but such a phenomenon was not observed in this study. Future research should aim to measure these constructs objectively, and to examine the extent to which reported preferences and enjoyment of activity translate into behaviour in situations where there is no environmental restriction, for example a laboratory free play situation. The sample size was modest, and some of the confidence intervals around the variance estimates relatively wide, so it is possible the study was underpowered to detect subtle genetic influences on objectively measured activity, especially in MVPA and sedentary behaviour where intraclass correlations were lower in DZ than MZ twins. Confidence in the results is increased by the large differences in estimates of heritability and shared environment between objectively measured activity and enjoyment of activity or fidgetiness, and the fact that genetic effects on objectively measured infant motor activity level have been detected in smaller samples than in the current study [14]; [48]. We plan to replicate these findings in a larger sample of twins in a future study [49]. It is also possible that if twins had been measured apart, genetic influence on accelerometery data would be stronger. However, we aimed for our objective measures to be reflective of *usual* activity behaviour, and it is likely that young twins, whether MZ or DZ, do spend a larger proportion of their time together. A number of participants were excluded because of instrument failure or lack of adherence, but there were no demographic differences between those who provided data or did not. Our sample was predominantly white (>90%) therefore the results may not be generalisable to other ethnic groups; although the TEDS sample is representative of families with twins in the UK [21]. Finally, the experience of growing up as a twin is undeniably different from other sibling relationships, but the levels of total physical activity, MVPA, and sedentary behaviour that we observed in this twin sample were very similar to data on non-twin children [50].

### Conclusions

These results indicate that even though there may be heritable differences in fidgetiness and enjoyment of activity, the shared environment can override expression of these genetic tendencies in actual day-to-day activity. This important finding supports the value of environmental interventions in childhood and highlights the need to identify the specific environmental factors that influence children's levels of activity.

## References

[1] Eisenmann JC, Tolfrey K (2008). Review of genetics and paediatric exercise science.. Pediatr Exerc Sci.

[2] Sallis RE (2009). Exercise is medicine and physicians need to prescribe it!. Br J Sports Med.

[3] Humpel N, Owen N, Leslie E (2002). Environmental factors associated with adults' participation in physical activity: a review.. Am J Prev Med.

[4] Sallis JF, Story M, Deborah L (2009). Study designs and analytic strategies for environmental and policy research on obesity, physical activity and diet.. Am J Prev Med.

[5] van Sluijs EM, McMinn AM, Griffin SJ (2007). Effectiveness of interventions to promote physical activity in children and adolescents: systematic review of controlled trials.. BMJ.

[6] Gorely T, Nevill ME, Morris JG, Stensel DJ, Nevill A (2009). Effect of a school-based intervention to promote healthy lifestyles in 7–11 year old children.. Int J Behav Nutr Phys Act.

[7] Webber LS, Catellier DJ, Lytle LA, Murray DM, Pratt CA (2008). Promoting physical activity in middle school girls: Trial of Activity for Adolescent Girls.. Am J Prev Med.

[8] McMurray RG, Harrell JS, Bangdiwala SI, Bradley C, Deng S (2002). A school-based intervention can reduce body fat and blood pressure in young adolescents.. J Adoles Health.

[9] Llewelly CH, van Jaarsveld CHM, Bonifance D, Carnell S, Wardle J (2008). Eating rate is a heritable phenotype related to weight in children.. Am J Clin Nutr.

[10] Plomin R, DeFries JC, McLean GE, McGuffin P (2008). Behavioural Genetics, 5th Edition..

[11] Stubbe JH, Boomsma DI, Vink JM, Cornes BK, Martin NG (2006). Genetic influences on exercise participation in 37,051 twin pairs from seven countries.. PLoS ONE.

[12] Duncan GE, Goldberg J, Noonan C, Moudon AV, Hurvitz P (2008). Unique environmental effects on physical activity participation: a twin study.. PLoS ONE.

[13] Stubbe JH, Boomsma DI, De Geus EJ (2005). Sports participation during adolescence: a shift from environmental to genetic factors.. Med Sci Sports Exerc.

[14] Saudino KJ, Eaton WO (1991). Infant temperament and genetics: an objective twin study of motor activity level.. Child Dev.

[15] Saudino KJ, Zapfe JA (2008). Genetic influences on activity level in early childhood: do situations matter?. Child Dev.

[16] Hwang J, Rothbart MK (2003). Behaviour genetics studies of infant temperament: findings vary across parent-report instruments.. Infant Behav Dev.

[17] Anderson SE, Bandini LG, Dietz WH, Must A (2004). Relationship between temperament, nonresting energy expenditure, body composition, and physical activity in girls.. Int J Obes Relat Metab Disord.

[18] Plomin R, Foch TT (1980). A twin study of objectively assessed personality in childhood.. J Pers Soc Psychol.

[19] Franks PW, Ravussin E, Hanson RL, Harper IT, Allison DB (2005). Habitual physical activity in children: the role of genes and the environment.. Am J Clin Nutr.

[20] Wood AC, Saudino KJ, Rogers H, Asherton P, Kuntsi J (2007). Genetic influences on mechanically-assessed activity level in children.. J Child Psychol Psychiatry.

[21] Oliver BR, Plomin R (2007). Twins' Early Development Study (TEDS): a multivariate, longitudinal genetic investigation of language, cognition and behavior problems from childhood through adolescence.. Twin Res Hum Genet.

[22] Wardle J, Guthrie C, Sanderson S, Birch L, Plomin R (2001). Food and activity preferences in children of lean and obese parents.. Int J Obes Relat Metab Disord.

[23] Reilly JJ, Penpraze V, Hislop J, Davies G, Grant S (2008). Objective measurement of physical activity and sedentary behaviour: review with new data.. Arch Dis Child.

[24] Ekelund U, Sjostrom M, Yngve A, Poortvleit E, Nilsson A (2001). Physical activity assessed by activity monitor and doubly labeled water in children.. Med Sci Sports Exerc.

[25] Andersen LB, Harro M, Sardinha LB, Froberg K, Ekelund U (2006). Physical activity and clustered cardiovascular risk in children: a cross-sectional study (The European Youth Heart Study).. Lancet.

[26] Mattocks C, Ness A, Leary S, Tilling K, Blair SN (2008). Use of accelerometers in a large field-based study of children: protocols, design issues, and effects on precision.. J Phys Act Health.

[27] Penpraze V, Reilly JJ, MacLean CMM, Montgomery C, Kelly LA (2006). Monitoring of physical activity in young children: how much is enough?. Pediatr Exerc Sci.

[28] Taylor E, Sandberg S (1984). Hyperactive behaviour in English schoolchildren: a questionnaire survey.. J Abnorm Child Psychol.

[29] Epstein L, Valoski A, Wing RR, Perkins KA, Fernstrom M (1989). Perception of eating and exercise in children as a function of child and parent weight status.. Appetite.

[30] Purslow LR, van Jaarsveld CH, Semmler C, Wardle J (2009). Validity and prognostic value of parental ratings of children's activity.. Prev Med.

[31] Cole TJ, Freeman JV, Preece MA (1995). Body mass index reference curves for the UK, 1990.. Arch Dis Child.

[32] Price TS, Freeman B, Craig I, Petrill SA, Ebersole L (2000). Infant zygosity can be assigned by parental report questionnaire data.. Twin Res.

[33] Thorburn AW, Proietto J (2000). Biological determinants of spontaneous physical activity.. Obes Rev.

[34] Wood AC, Rijsdijk F, Saudino KJ, Asherson P, Kuntsi J (2008). High heritability for a composite index of children's activity level measures.. Behav Genet.

[35] Auerbach JG, Faroy M, Ebstein R, Kahana M, Levine J (2001). The association of the dopamine D4 receptor gene (DRD4) and the serotonin transporter promoter gene (5-HTTLPR) with temperament in 12-month-old infants.. J Child Psychol Psychiatry.

[36] Kiang M, Daskalaksis J, Christensen BK, Remington G, Kapur S (2003). Actigraphic measurement of the effects of single-dose haloperidol and olanzapine on spontenous motor activity level in normal subjects.. J Psychiatry Neuropsych.

[37] Levine JA (2004). Non-exercise activity thermogenesis (NEAT).. Nut Rev.

[38] Levine JA, Eberhardt NL, Jensen MD (1999). Role of nonexercise activity thermogenesis in resistance to fat gain in humans.. Science.

[39] Johannsen DL, Ravussin E (2008). Spontaneous physical activity: relationship between fidgeting and body weight control.. Curr Opin Endocrinol Diabetes Obes.

[40] Fox PW, Hershberger SL, Bouchard TJ (1996). Genetic and environmental contributions to the acquisition of a motor skill.. Nature.

[41] Roemmich JN, Barkley JE, Lobarinas CL, Foster JH, White TM (2008). Association of liking and reinforcing value with children's physical activity.. Physiol Behav.

[42] Simonen RL, Rankinen T, Perusse L, Leon AS, Skinner JS (2003). A dopamine D2 receptor gene polymorphism and physical activity in two family studies.. Physiol Behav.

[43] Epstein LH, Smith JA, Vara LS, Rodefer JS (1991). Behavioral economic analysis of activity choice in obese children.. Health Psychol.

[44] Ekelund U, Yngve A, Brage S, Westereterp K, Sjostrom M (2004). Body movement and physical activity energy expenditure in children and adolescents: how to adjust for differences in body size and age.. Am J Clin Nutr.

[45] Esliger DW, Tremblay MS, Copeland JL, Barnes JD, Huntington GE (2010). Physical activity profile of Old Order Amish, Mennonite, and contemporary children.. Med Sci Sports Exerc.

[46] Neale MC, Stevenson J (1989). Rater bias in the EASI temperament scales: a twin study.. J Pers Soc Psychol.

[47] Saudino KJ, Cherny SS, Plomin R (2000). Parent ratings of temperament in twins: explaining the ‘too low’ DZ correlations.. Twin Res.

[48] Saudino KJ, Eaton WO (1995). Continuity and change in objectively assessed temperament: a longitudinal twin study of activity level.. Br J Dev Psychol.

[49] van Jaarsveld CH, Johnson L, Llewellyn C, Wardle (2010). J Gemini: a UK twin birth cohort with a focus on early childhood weight trajectories, appetite and the family environment.. Twin Res Hum Genet.

[50] Purslow LR, Hill C, Saxton J, Corder K, Wardle J (2008). Differences in physical activity and sedentary time in relation to weight in 8–9 year old children.. Int J Behav Nutr Phys.

